# Analysis and validation of characteristic genes in RNA sequencing datasets from heart failure patients based on multiple algorithms

**DOI:** 10.3389/fcvm.2025.1559429

**Published:** 2025-08-26

**Authors:** Yuxuan Li, Ying Bai, Wujiao Wang, Zhaotian Ma, Peng Li, Dong Li, Sinai Li, Jialin Jin, Qian Lin

**Affiliations:** ^1^ Department of Cardiology, Dongzhimen Hospital, Beijing University of Chinese Medicine, Beijing, China; ^2^ Department of Traditional Chinese Medicine, Peking Union Medical College Hospital, Beijing, China; ^3^ College of Pharmacy, Jining Medical University, Jining, China; ^4^ Department of Cardiology, Dongfang Hospital, Beijing University of Chinese Medicine, Beijing, China; ^5^ Beijing Hospital of Traditional Chinese Medicine, Capital Medical University, Beijing Institute of Traditional Chinese Medicine, Beijing, China; ^6^ China Science and Technology Development Center for Chinese Medicine, Beijing, China

**Keywords:** heart failure, myocardial tissue, transcriptome, weighted correlation network analysis, bioinformatics analysis, gene set enrichment analysis, single-cell transcriptome

## Abstract

**Background:**

Patients with heart failure (HF) have a poor prognosis and continue to pose a global threat to human health. Consequently, it is crucial to employ bioinformatic approaches to analyze functional alterations within the transcriptome. This analysis should be conducted in conjunction with transcriptome sequencing data from a large sample of clinical myocardial tissue, in order to identify the core pathogenic mechanisms in heart failure myocardial tissue.

**Method:**

Transcriptome data from HF patient myocardial biopsies underwent Robust Rank Aggregation (RRA) to identify differentially expressed genes (DEGs). These DEGs were intersected with key genes identified via Weighted Gene Co-expression Network Analysis (WGCNA) in HF. Functional enrichment analysis was performed on the DEGs. Selected key genes were experimentally validated using RT-qPCR in hypertrophic cardiomyocyte models. Single-cell data dimensionality reduction, clustering, and visualization were achieved using Principal component analysis (PCA) and uniform manifold approximation and projection (UMAP). Cell types were annotated with SingleR and CellMarker, and single-cell functional enrichment was performed using the “irGSEA” R package.

**Results:**

RRA of transcriptome data from five studies identified 102 DEGs. Functional enrichment analyses (GO, KEGG, GSEA) revealed associated functional alterations. WGCNA highlighted a key module enriched for energy metabolism-related genes, with the mitochondrial matrix and inner membrane identified as their primary subcellular locations. Integrating RRA-derived DEGs with WGCNA key module genes yielded 14 crucial genes, validated experimentally in a hypertrophic cardiomyocyte model. Analysis of single-cell RNA-seq data identified cold shock domain containing C2 (CSDC2) and Single-pass membrane and coiled-coil domain-containing protein 4 (SMCO4) as cardiomyocyte-specific genes within this set. Subpopulations of cardiomyocytes with high or low expression of SMCO4 and CSDC2 showed strong associations with alterations in fatty acid metabolism, adipogenesis, and oxidative phosphorylation pathways.

**Conclusion:**

Integrated transcriptomic analysis identified 12 key genes linked to HF, which were validated in a hypertrophy model. Single-cell data showed SMCO4 and CSDC2 are specifically expressed in cardiomyocytes and regulate fatty acid metabolism. This suggests SMCO4 and CSDC2 contribute to HF by altering fatty acid metabolism in heart cells, revealing new disease mechanisms.

## 1 Introduction

Heart failure(HF) poses a significant threat to human health on a global scale. Although important advances have been made in the treatment of HF in recent years, breakthroughs in pharmacotherapy have primarily been in diuretic and vasodilatory treatments that reduce systemic circulation load, thereby providing comprehensive benefits for patients. The search for new therapeutic targets to further reduce patient mortality remains a hot topic of research.

Basic research has revealed a significant correlation between mitochondrial function and pathological hypertrophy of cardiomyocytes (1, 2). In hypertrophied cardiomyocytes, increased mitochondrial fission and activated mitophagy are observed (3), with mice exhibiting severe cardiac dysfunction and structural alterations (4). Cardiomyocytes require mitochondria to generate substantial ATP for their enormous energy demands during contraction and relaxation. Fatty acids and glucose serve as the heart's primary substrates, each possessing distinct energy-yielding advantages. Fatty acids constitute the dominant cardiac fuel, with fatty acid oxidation accounting for 40%–60% of myocardial ATP production (5). While healthy hearts maintain flexible switching between fatty acid and glucose metabolism to sustain oxidative phosphorylation and ATP generation (6), HF triggers metabolic reprogramming that shifts substrate preference toward glycolysis. This transition from fatty acid to glucose metabolism proves detrimental since glycolysis yields far less ATP than oxidative phosphorylation, ultimately causing energy deficiency (7). Consequently, investigating HF pathogenesis through the lens of mitochondrial function and metabolic changes—particularly in fatty acid metabolism—holds significant clinical relevance for identifying therapeutic targets.

HF originates from pathological factors that alter multiple genes, involving gene mutations and complex pathophysiological processes (8). By employing bioinformatics techniques to analyze transcriptomic data, it is possible to perform massive computations to parse and predict gene changes, thereby revealing complex pathogenic mechanisms. This is of significant importance for exploring therapeutic targets for HF.

## 2 Materials and methods

### 2.1 Data source and processing

The sample sequencing datasets were selected according to the following criteria: Studies were retrieved from the Gene Expression Omnibus (GEO) database (https://www.ncbi.nlm.nih.gov/geo/) using “HF” as the MeSH term, with the organism filter set to *Homo sapiens* and the entry type restricted to series. The study types were limited to expression profiling by array and expression profiling by high-throughput sequencing. The tissue attribute was specified as left ventricular tissue. Through the screening process illustrated in the flow diagram (Supplementary Figure S1), six specific datasets (GSE57338, GSE42955, GSE52601, GSE21610, GSE76701, and GSE145154) were selected for inclusion in our study (Table 1) (9–14). For detailed clinical information of the GEO datasets, see Supplementary Table S1. Subsequently, the gene expression profiles and corresponding clinical information were downloaded for further analysis. Probe data from different platforms were preprocessed using the R packages tidyverse, affy, and annotationDbi for gene symbol conversion, removal of duplicate values, and handling of missing values. The limma package was utilized for normalization and batch effect correction. Additional batch effects were mitigated using the FactoMineR and factoextra packages. For single-cell transcriptome sequencing, the Seurat package in R was employed for data importation, quality control, filtering, normalization, selection of cell type-specific features, and extraction of cardiomyocyte expression profiles. Data quality control was based on gene counts per cell being >200 and <2,500, along with a mitochondrial gene percentage <20%. Subsequently, the “vst” method was applied in the FindVariableFeatures function to select the top 2,000 highly variable genes. Principal component analysis (PCA) and data clustering analysis were performed using uniform manifold approximation and projection (UMAP) via the RunPCA, FindClusters, and RunUMAP functions. Additionally, we utilized Single R V1.4.1 and CellMarker 2.0 for cell type annotation (15). Finally, single-cell functional enrichment analysis was conducted using the R package “irGSEA”. We obtained fatty acid metabolism-related genes from MSigDB (https://www.gsea-msigdb.org/gsea/msigdb/), which contains hallmark gene sets. A total of 158 genes were identified for further analysis.

**Table 1:** GEO dataset basic information.

No.	GEO ID	Heart failure	Normal	Genes	Sequencing platform
1	GSE57338	177	136	18,851	Affymetrix Human Gene 1.1 ST Array
2	GSE52601	8	4	13,713	Illumina HumanHT-12 V4.0 expression beadchip
3	GSE42955	24	5	18,851	Affymetrix Human Gene 1.0 ST Array
4	GSE21610	30	8	20,824	Affymetrix Human Genome U133 Plus 2.0 Array
5	GSE76701	4	4	20,824	Affymetrix Human Genome U133 Plus 2.0 Array
6	GSE145154	18	4	58,233	GPL20795 HiSeq X Ten
					GPL24676 Illumina NovaSeq 6000

All samples are human ventricular myocardial tissues.

### 2.2 Identifying differential genes

After obtaining the gene expression matrix of the dataset in a common format, the Bioconductor package and the limma package were used to perform Bayesian multiple testing correction methods to calculate the differential changes between clinical grouping data; the threshold for significant differences was set at *P* value < 0.05 & |LogFC| > 0.3.

As a statistical algorithm for integrating prioritized gene lists from multiple datasets, Robust Rank Aggregation (RRA) identifies genes that are consistently ranked better than expected under the null hypothesis of random input lists. It assigns significance scores via order statistics to compare observed ranks with random expectations (16). Distinct from other algorithms, RRA is parameter-free, robust to noise/outliers, handles partial rankings, and efficiently computes statistically rigorous significance scores—unlike average rank (noise-sensitive) or the Stuart method (computationally expensive with non-*P*-value scores).

To minimize cross-study dependence, our study selected five GEO datasets derived from independent experiments and distinct cohorts. Each dataset underwent independent processing and normalization prior to gene list ranking, ensuring inter-dataset comparability.Subsequently, the “RobustRankAggreg” package was employed to integrate and analyze these ranked gene lists. This method internally validates ranking consistency using order statistics under the null hypothesis of random ordering, thereby ensuring statistical rigor.

### 2.3 Weighted gene co-expression network analysis

The WGCNA is an R package for constructing and analyzing weighted correlation networks, particularly focused on gene co-expression networks. It identifies clusters (modules) of highly correlated genes, summarizes modules using eigengenes or intramodular hub genes, relates modules to external sample traits (e.g., disease status), and quantifies gene-module relationships via fuzzy module membership measures (17). After excluding outlier samples, we clustered to obtain different gene modules. Subsequently, we used the pickSoftThreshold function in R to select a soft threshold (*β*). Based on the *β* value, we calculated the adjacency matrix and then used the TOMsimilarity function to obtain the topological overlap matrix. Dynamic tree cutting cluster analysis was applied to acquire gene modules with similar expression patterns. Functional enrichment was conducted on key modules, followed by intersection analysis with RRA differential genes. The intersecting genes are considered to be key differential genes in HF.

### 2.4 Gene set enrichment analysis

GSEA focuses on the collective analysis of genes, ranking all genes based on their differential expression fold changes. It then compares these rankings to existing gene function databases, aligning known gene sets to the entire gene ranking to determine the trend of changes in these gene sets under disease conditions. GSEA can be implemented using the clusterProfiler package functions in R. The specific process involves formatting the data, converting gene symbols to entrezid format, ranking genes by fold change, downloading the GSEA database GMT file, and analyzing and plotting the results.

### 2.5 Gene functional enrichment analysis

Gene functional enrichment can reveal the changes in biological functions underlying bioinformatics differences. To evaluate the biological processes (BP), molecular functions (MF), cellular components (CC), and signaling pathways associated with the target gene expression profiles obtained from various analysis methods, GO and KEGG functional enrichments are performed (18, 19). This can be achieved using the enrichGO and enrichKEGG functions from the clusterProfiler package, along with the org.Hs.eg.db and AnnotationDbi packages in R. The underlying mathematical principle involves hypergeometric distribution/Fisher's exact test. The false discovery rate (FDR) is controlled using the Benjamini-Hochberg procedure, with a selection criterion of FDR < 0.05. The relevant plotting is carried out using the ggplot2 package in R.

### 2.6 RT-qPCR

We employed the Trizol method to extract total RNA, and the collected RNA was placed on ice and then transferred to a spectrophotometer (Nanodrop 2,000) to assess concentration and quality. Subsequently, reverse transcription was performed to synthesize cDNA, and quantitative real-time PCR (qRT-PCR) was conducted using a fluorescence-based PCR instrument (Roche LightCycler480) to detect mRNA expression. The PCR reaction conditions were as follows: set at 95°C for 15 min for 1 cycle, and 95°C for 10 s, 60°C for 32 s for 40 cycles; to calculate the relative gene expression levels in each sample, we utilized the cycle threshold method (2−*ΔΔ*Ct). GAPDH was used as the internal control gene. Detailed information on the primer sequences can be found in the Table 2.

**Table 2:** Primer sequence table.

Name	Forward (5′-3′)	Reverse (5′-3′)
PDK4	CATCCTCCCTGAACGCTTAGTGAAC	TTTCTGGTCTTCTGGGCTCTTTTCG
SCN2B	TCATCGTGGGTGCCTCAGTGG	GTCTTGCCTTCCTCTTCGGTCTTC
CHDH	CATCCAGTTCCACTTCCTGCCATC	CTCAGTTTCAGCCAGCCCACAC
GAPDH	ACGGCAAGTTCAACGGCACAG	CGACATACTCAGCACCAGCATCAC
SERPINA3	CTGCCTTAGTCCTCCTGTCCCTAG	TCATAGCCCTGGTGGATCTCTGC
BCL6	GAGGTCGTGAGGTTGTGGAGAAC	TCGGATAAGAGGCTGGTGGTGTC
BID	CACAGCATCCAGCCCACACTG	GCCTTGTCATTCTCCATGTCCCTAG
SCGN	AGCATCAGCGGTGTGGATCTTG	TTCCATCCTTGTTCACATCGCAATG
BLM	ACAGAAGTACGCAGAGTGGACATTG	GCTTCCTCCTCCTCCGTCTCC
FCN3	TTGAGTGGCTGGTATCGCTTGTG	CACCTCCTGTACTGTCCATGTCAC
PLIN2	CTCCACTCCACTGTCCATCTGATTG	GTAGCCGACGATTCTCTTCCACTC
CSDC2	CTACCAAACGGACCAGGACATACTC	TGCTTACAGACGCCCTTGAACAC
SMCO4	CCAAGGACAAGAAGGAGCGGAAG	CTACAATGAGGAGCACCACCACAG
TMTC1	CAAGAGGCTGGAGCACAAGGAAG	AAGAAGAGGTTGCTGGCTGGAATG
GALNT15	GGATTGGAGGACAGAGGAGGATGG	GGCACTGAGTGGCTTGTTGAGG

### 2.7 Neonatal rat primary cardiomyocyte hypertrophy model

Neonatal rat primary cardiomyocytes (NRCMs) were isolated from the hearts of 1- to 3-day-old male Sprague-Dawley rats. The rats were provided by Beijing Vital River Laboratory Animal Technology Co., Ltd. (SCXK Beijing 2021-0011). The entire research adhered to the principles of laboratory animal science and was approved by the Animal Ethics Committee of the Basic Research Institute, China Academy of Chinese Medical Sciences, with the ethical approval number (ERCCACMS 21-2406-02). The NRCMs were placed in a CO2 incubator to differentially adhere and enrich cardiomyocytes. After 1.5 h, the supernatant was collected to obtain a primary cardiomyocyte suspension, and the cell concentration was adjusted to 5 × 105/ml. The cells were then plated into culture plates or flasks as required, and incubated in the incubator under the same conditions for further culture. After 24 h of culture, the cells were used for experiments. A hypertrophy model of cardiomyocytes was prepared by stimulating the cells with angiotensin II (1 μM) (20).

### 2.8 Statistical analysis

All statistical analysis was performed using R (version4.2.3) and associated R packages. A significance level of *P* < 0.05 was used for all analyses to indicate statistical significance.

## 3 Results

### 3.1 Identification of key differential genes and functional enrichment analysis

**Figure 1:**
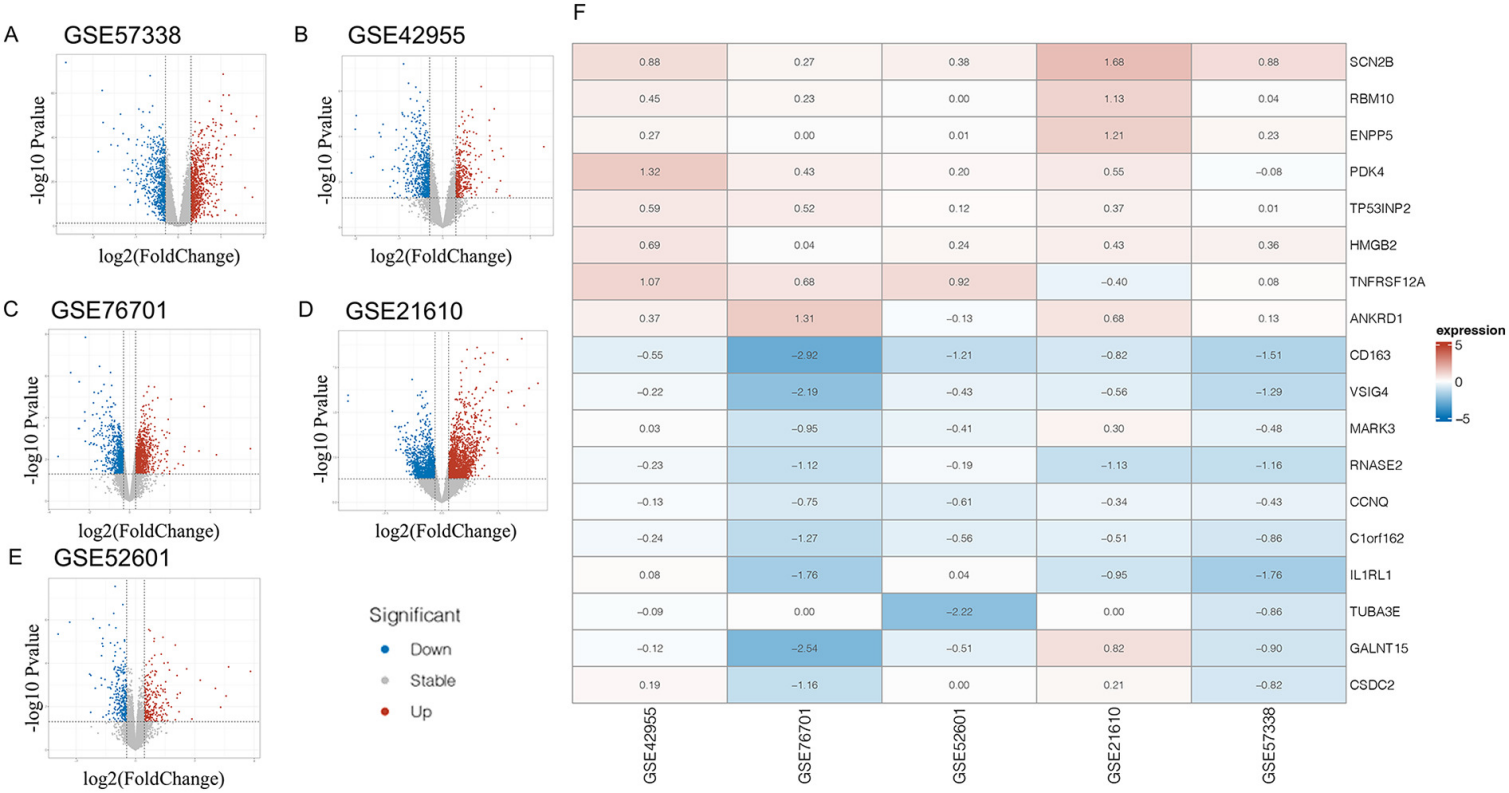
Panels **(A–E)** display volcano plots of differentially expressed genes (DEGs) across five independent heart failure transcriptomic datasets. Panel **(F)** shows a heatmap of the top 10 upregulated and downregulated DEGs identified through Robust Rank Aggregation (RRA) analysis, which integrates ranked gene lists from multiple studies.

Individual differential analyses were conducted on the GSE57338, GSE42955, GSE52601, GSE21610, and GSE76701 datasets (Figures 1A–E). Subsequently, using RRA with |LogFC| > 0.3 as the screening criterion, 913 differential genes were obtained. Since the joint analysis of multiple datasets increases its robustness, these genes should be included in subsequent analyses and are referred to as RRA trend genes. When using *P* value <0.05 & |LogFC| > 0.3 as the screening criteria, a total of 102 differential genes (DEGs) were obtained, consisting of 8 upregulated genes and 94 downregulated genes. These genes are referred to as RRA differential genes, and the heatmap displayed the genes with the top differential magnitudes (Figure 1F). The threshold of |LogFC| > 0.3 was selected to capture more subtle yet biologically meaningful transcriptional differences in myocardial tissue, particularly considering that mitochondrial or metabolic genes often exhibit moderate fold changes—a practice that is permissible in numerous studies (21–23). A more stringent threshold (e.g., |LogFC| > 1) would likely miss relevant genes with small but consistent changes across datasets. The *p*-value < 0.05 cutoff is standard and ensures statistical significance.

**Figure 2:**
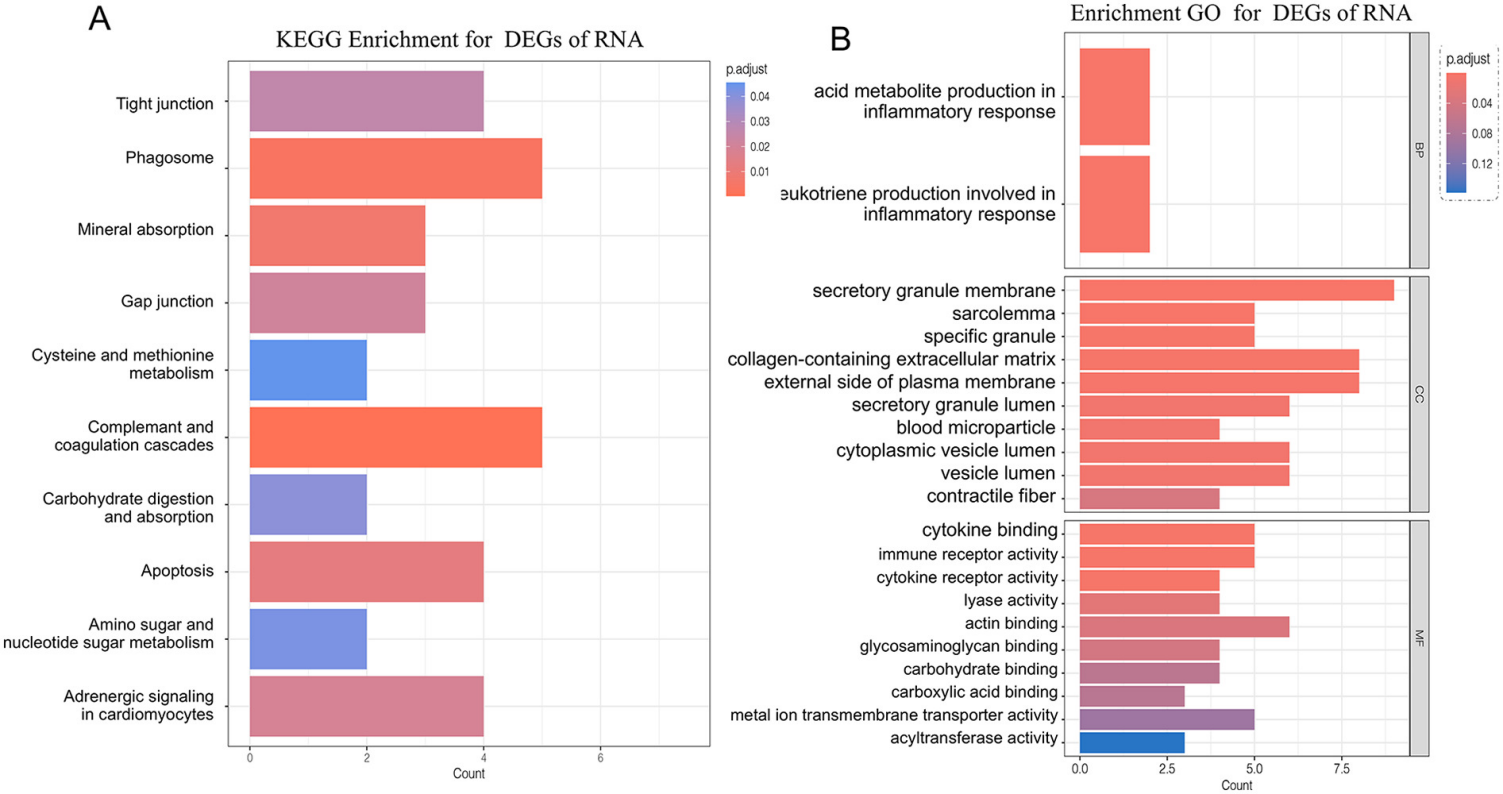
Panel **(A)** shows the KEGG pathway enrichment analysis of DEGs identified through RRA. Panel **(B)** displays the gene ontology (GO) functional enrichment analysis of RRA-derived DEGs, categorized into biological processes, molecular functions, and cellular components.

To thoroughly understand the changes in biological functions associated with RRA differential genes, we conducted GO and KEGG enrichment analyses (Figures 2A,B). The GO functional enrichment analysis revealed that RRA differential genes were enriched in 2 genes involved in the biological processes of arachidonic acid metabolite production involved in inflammatory response and leukotriene production involved in inflammatory response.

In terms of cellular components, enrichment was observed in secretory granule membrane, collagen-containing extracellular matrix, external side of the plasma membrane, cytoplasmic vesicle lumen, secretory granule lumen, vesicle lumen, sarcolemma, specific granule, blood microparticle, and contractile fiber locations.

In molecular function, RRA differential genes were primarily enriched in actin binding, cytokine binding, immune receptor activity, metal ion transmembrane transporter activity, carbohydrate binding, cytokine receptor activity, glycosaminoglycan binding, lyase activity, acyltransferase activity, and carboxylic acid binding.

KEGG analysis showed that genes were involved in the complement and coagulation cascades, phagosome pathway, mineral absorption pathway, apoptosis pathway, adrenergic signaling in cardiomyocytes pathway, gap junction pathway, tight junction pathway, carbohydrate digestion and absorption pathway, amino sugar and nucleotide sugar metabolism pathway, and cysteine and methionine metabolism pathway.

### 3.2 Gene set enrichment analysis

Since the functional enrichment analysis of differential genes did not show a significant trend and covered a wide range of cellular life activities, a GSEA was subsequently conducted to thoroughly examine the RRA trend genes. Among the RRA trend genes (913 genes), not all gene changes were statistically significant. However, considering that the joint analysis of the five datasets increased the robustness of these 913 genes, to reduce the loss of data due to statistical rules, GSEA analysis was used to reflect the genes with common regulatory trends and their functional changes across the five datasets.

**Figure 3:**
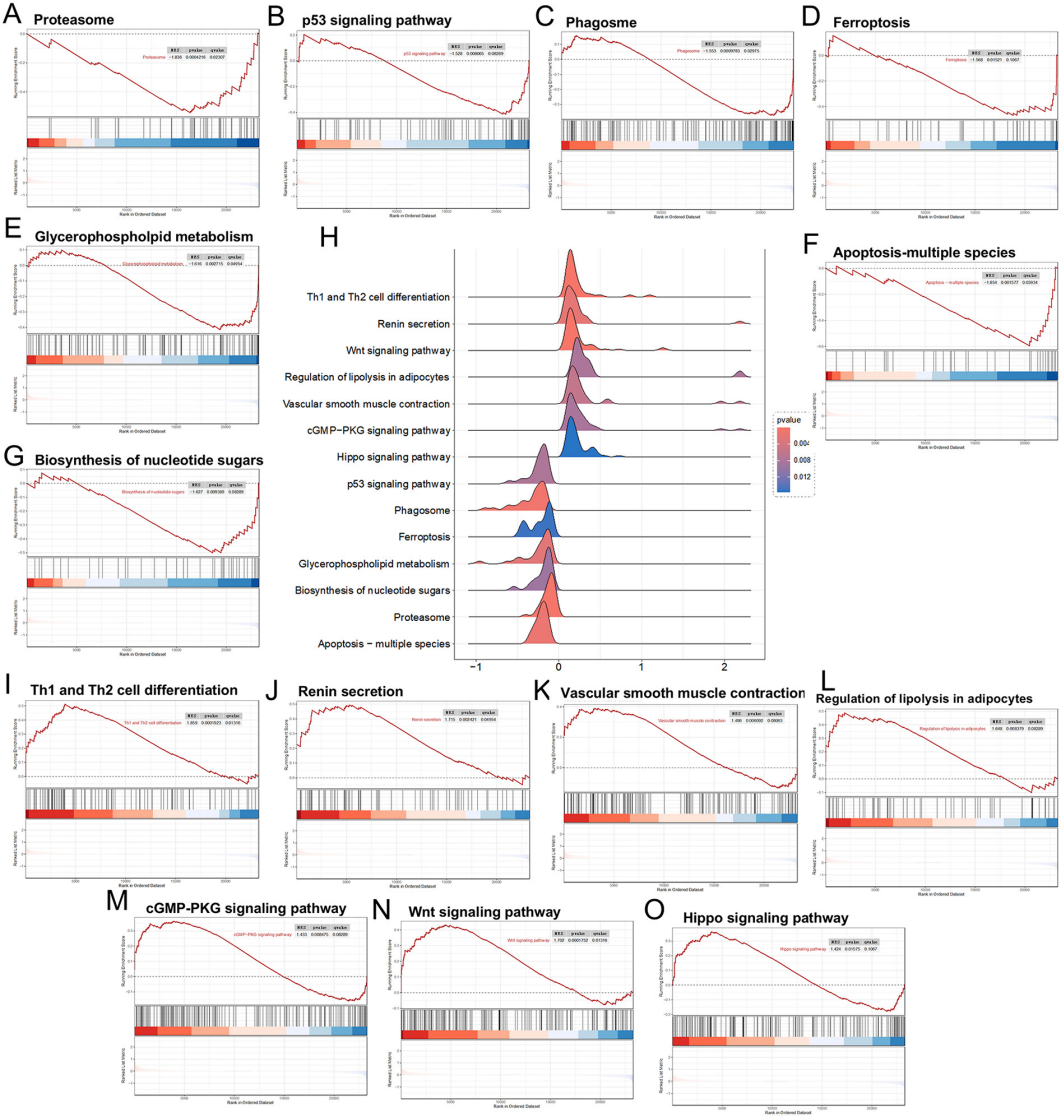
Panels **(A–G)**, downregulated differential genes in the GSEA analysis; **(H)** landscape plot of GSEA results; **(I–O)** upregulated differential genes in the GSEA analysis.

Among the RRA trend genes, the downregulated biological functions included the Proteasome, P53 signaling pathway, Phagosome, Ferroptosis, Glycerophospholipid metabolism, Biosynthesis of nucleotide sugars, and Apoptosis—multiple species (Figures 3A–G). The upregulated biological functions included Th1 and Th2 cell differentiation, Renin secretion, Wnt signaling pathway, Regulation of lipolysis in adipocytes, Vascular smooth muscle contraction, cGMP−PKG signaling pathway, and Hippo signaling pathway (Figures 3H–O).

In summary, the GSEA results still covered a wide range of cellular life activities, and the aspects involved were functionally consistent with the pathological changes in myocardial tissues of HF, but no obvious trend was observed.

### 3.3 Construction and identification of key modules in the WGCNA network for HF

**Figure 4:**
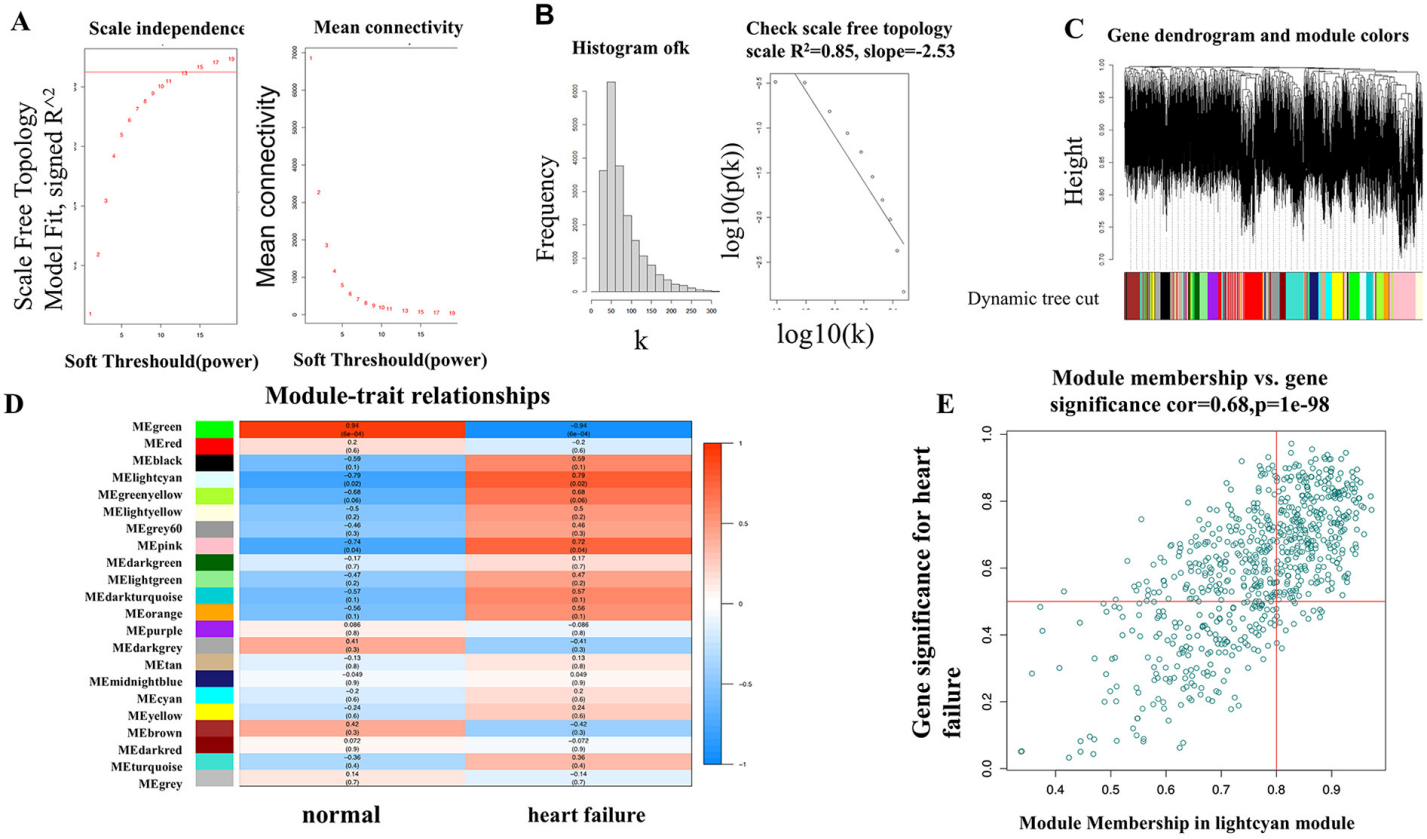
**(A,B)** Selection of the soft threshold in WGCNA analysis; **(C)** modular partitioning of the overall expression matrix for the GSE76701 dataset; **(D)** association analysis between gene modules and heart failure phenotypes for the GSE76701 dataset; **(E)** Scatter plot of the association between genes in the lightcyan module and heart failure phenotypes.

When constructing a WGCNA network, it is necessary for the data to conform as closely as possible to a scale-free distribution. After performing power calculations on the five datasets, it was determined that the GSE76701 dataset had the distribution closest to a scale-free distribution (*R*^2^ = 0.84) (Figures 4A,B). Dynamic tree cutting cluster analysis was applied to obtain gene modules with similar expression patterns (Figure 4C), with different colors used to distinguish between modules. A total of 22 gene modules were obtained after dynamic cutting and merging of similar modules, and specific module information can be found in Table 3.

**Table 3:** The number of genes in each module of the GSE76701 dataset.

Gene module	Gene count	Gene module	Gene count
Green	1,015	Orange	489
Red	2,470	Purple	833
Black	960	Darkgrey	509
Lightcyan	722	Tan	819
Greenyellow	820	Midnightblue	755
Lightyellow	657	Cyan	786
Grey60	670	Yellow	1,032
Pink	3,063	Brown	1,064
Darkgreen	607	Darkred	622
Lightgreen	664	Turquoise	1,607
Darkturquoise	598	Grey	65

We visualized the correlation between each gene module and HF phenotypes (Figure 4D). The results showed that the gene module with the highest correlation with the HF disease phenotype was the lightcyan module (correlation coefficient 0.79, *P* = 0.02), which contains 722 genes. We presented the correlation between these genes and the HF phenotype using a scatter plot. It can be observed that as the gene expression in the lightcyan module increases, the correlation between the gene dots and the HF phenotype significantly increases in a positively correlated linear relationship (Figure 4E).

**Figure 5:**
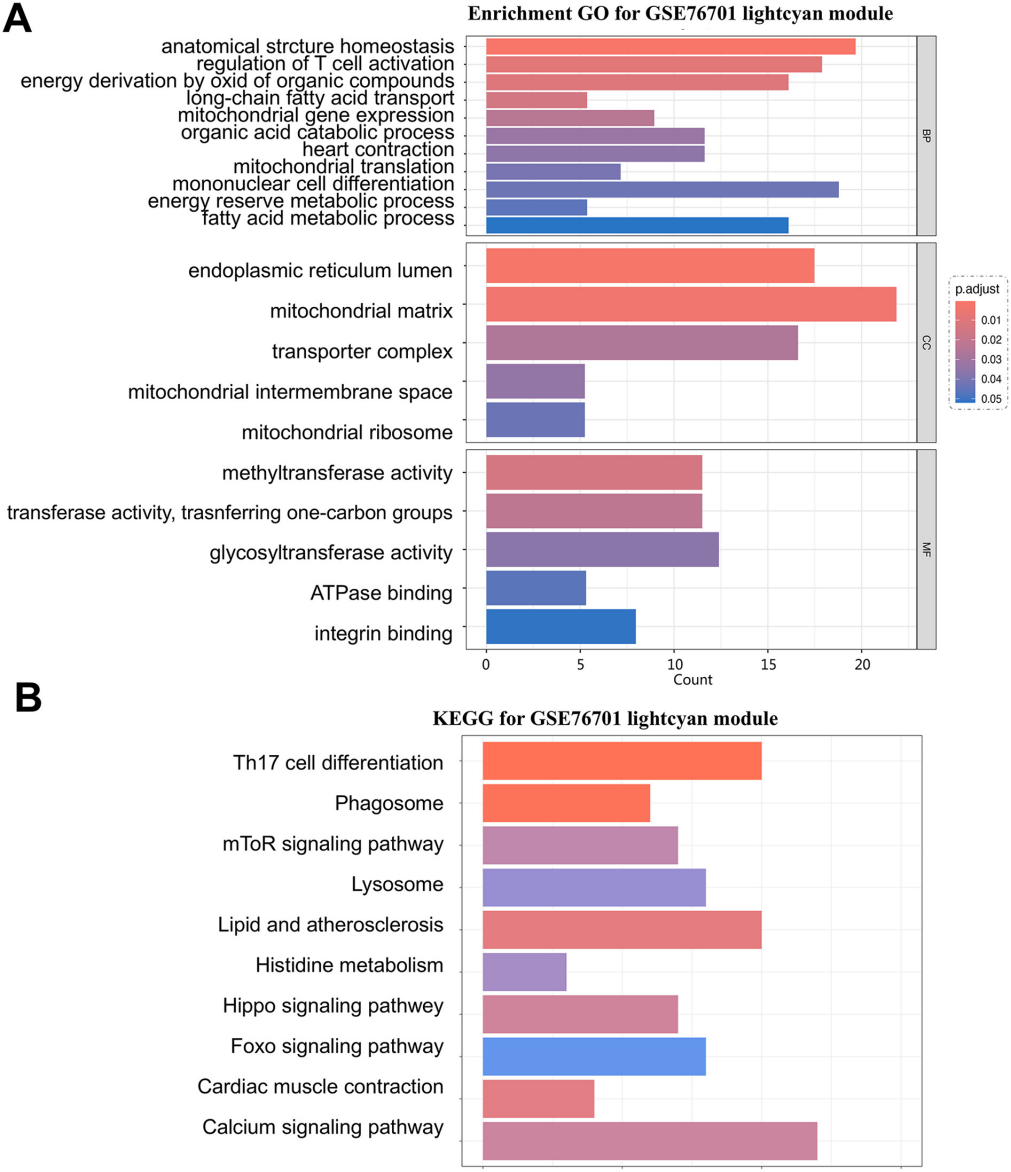
**(A)** GO analysis of the lightcyan module in the GSE76701 dataset; **(B)** representative results of KEGG analysis for the key module in the GSE76701 dataset.

The lightcyan module genes are the most highly associated gene modules with HF in the WGCNA analysis. Functional analysis of this module can uncover key pathogenic mechanisms of HF. Following GO and KEGG analyses, the results indicate a certain propensity towards energy metabolism. The GO analysis is primarily enriched in biological functions such as energy derivation by oxidation of organic compounds, fatty acid metabolic process, mitochondrial gene expression, long-chain fatty acid transport, and energy reserve metabolic process, and in terms of cellular components, it highlights the mitochondrial matrix and mitochondrial intermembrane space as the main locations where key genes are primarily localized (Figure 5A). The KEGG analysis also enriches in functional pathways related to energy cycle utilization, such as the mTOR signaling pathway, phagosome signaling pathway, and lysosome signaling pathway (Figure 5B).

### 3.4 Intersection analysis of RRA and WGCNA differential genes and validation by RT-qPCR

**Figure 6:**
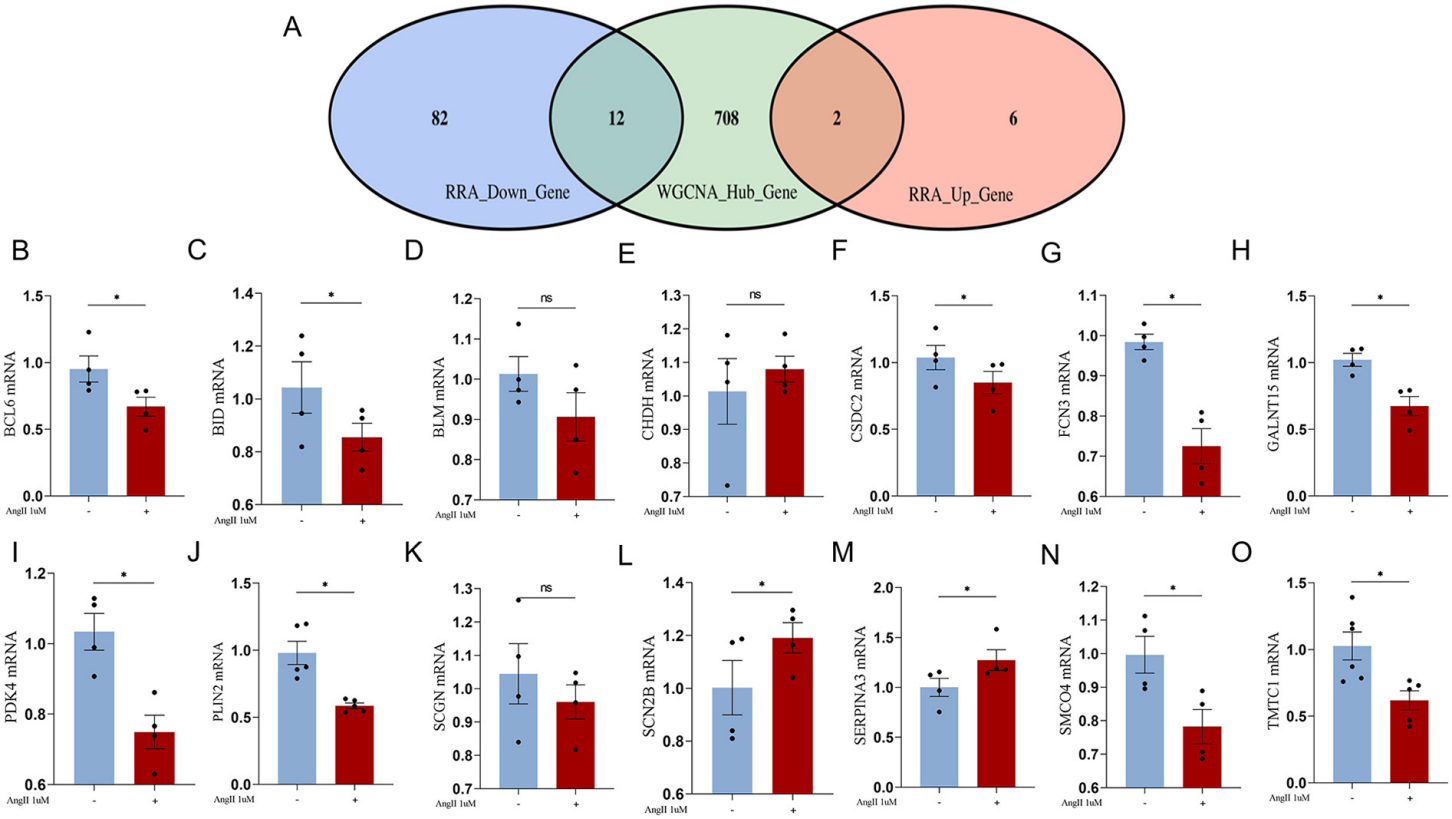
**(A)** Venn diagram of RRA differential genes intersecting with WGCNA lightcyan module genes; **(B–O)**, RT-qPCR validation of the intersection genes between RRA differential genes and WGCNA lightcyan module genes.

The intersection analysis of RRA differential genes and WGCNA module analysis uncovered pathogenic genes highly associated with HF from two perspectives. The intersection of RRA differential genes with key module genes from WGCNA, followed by validation of the expression of the intersecting genes using the AngII-induced primary rat cardiomyocyte hypertrophy model, is of significant importance. A total of 14 genes were obtained after the intersection (Figure 6A) (Table 4).

**Table 4:** The intersection genes of RRA differential genes and key module genes from WGCNA.

ID	Upregulated gene	Gene description	Downregulated gene	Gene description
1	PDK4	Pyruvatedehydrogenasekinase4	CHDH	Cholinedehydrogenase
2	SCN2B	Sodiumvoltage-gatedchannelbetasubunit2	SERPINA3	SerpinfamilyAmember3
3	\	\	BCL6	BCL6transcriptionrepressor
4	\	\	BID	BH3interactingdomaindeathagonist
5	\	\	SCGN	Secretagogin
6	\	\	BLM	BLMRecQlikehelicase
7	\	\	FCN3	Ficolin3
8	\	\	PLIN2	Perilipin2
9	\	\	CSDC2	ColdshockdomaincontainingC2
10	\	\	SMCO4	Single-PassMembraneProteinWithCoiled-CoilDomains4
11	\	\	TMTC1	TransmembraneO-mannosyltransferasetargetingcadherins1
12	\	\	GALNT15	PolypeptideN-acetylgalactosaminyltransferase15

We constructed an AngII-induced primary rat cardiomyocyte hypertrophy model to simulate the pathological changes in HF myocardial tissue. The expression of the aforementioned intersecting genes were verified by RT-qPCR. Among the upregulated genes, the expression of SCN2B gene increased, which is consistent with the trend predicted by bioinformatics analysis (Figure 6L). The expression trend of PDK4 gene was opposite to the prediction (Figure 6I). Among the downregulated genes, the expression of BCL6, BID, CSDC2, FCN3, GALNT15, PLIN2, SMCO4, and TMTC1 genes decreased, which is consistent with the predicted trend. The expression trend of SERPINA3 gene was opposite to the prediction. The changes in BLM, CHDH, and SCGN genes were not statistically significant, but the trends were consistent with the predictions (Figures 6B–O).

### 3.5 CSDC2 and SMCO4 may influence the progression of HF by modulating fatty acid metabolism in cardiomyocytes

**Figure 7:**
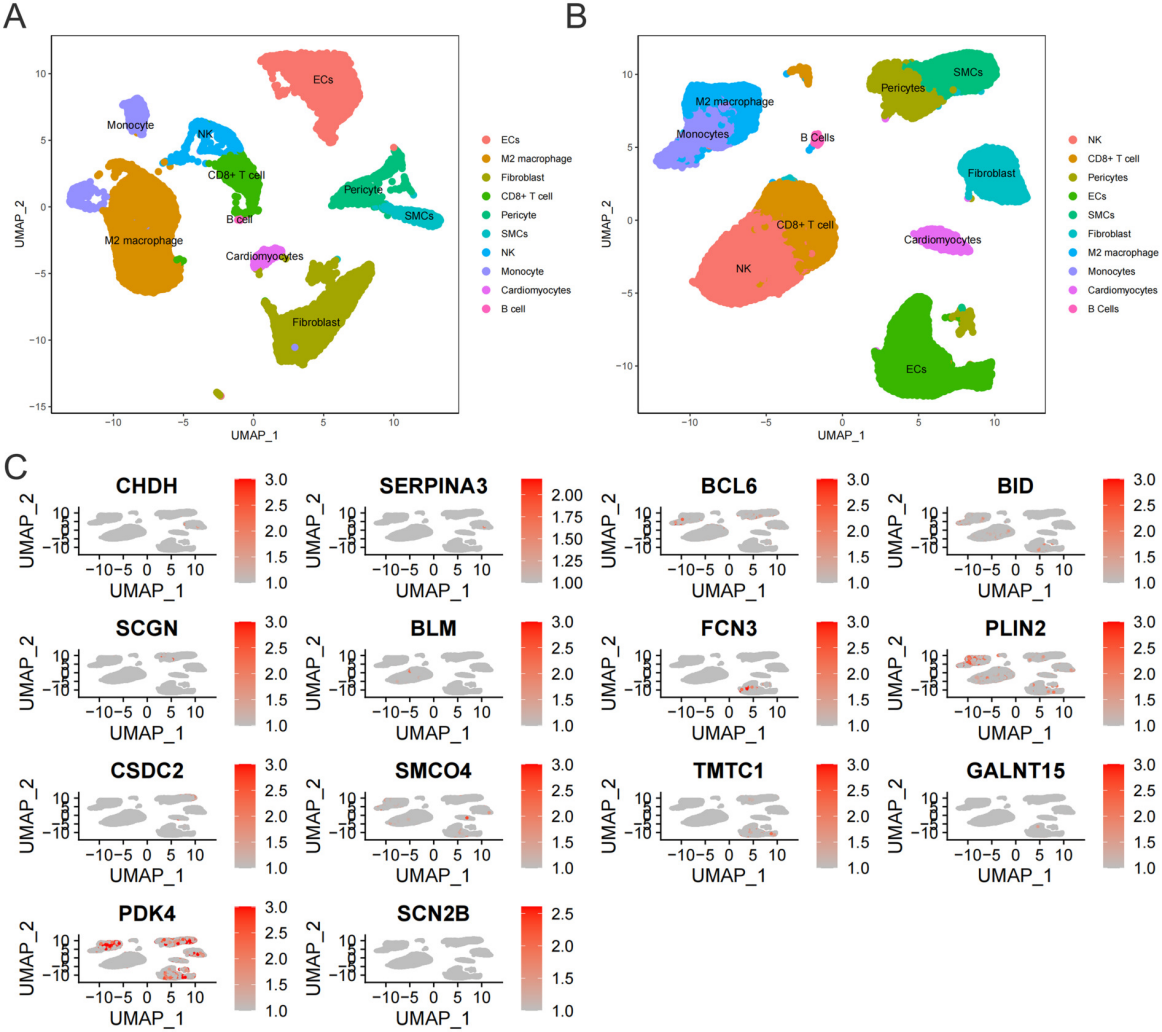
**(A)** Cell subtypes in normal myocardial tissue. **(B)** Cell subtypes in myocardial tissue of HF patients. **(C)** Expression of 14 core genes in myocardial tissue of HF patients.

The single-cell sequencing data in the GSE145154 dataset were obtained from ventricular tissues. Through PCA and UMAP, similar cells were clustered into the same subgroup. Both normal myocardial tissue and the myocardial tissue in the HF group showed that the cell populations were divided into 10 types: Monocytes, Natural Killer (NK) cells, Fibroblasts, M2 macrophages, Cardiomyocytes, B cells, Endothelial cells (ECs), Cluster of Differentiation 8 positive T cells (CD8^+^ T cells), Pericytes, and Smooth muscle cells (SMCs) (Figures 7A,B).

To experimentally validate the association between the 14 key genes and cardiomyocytes in a real-world context, we selected 11 genes exhibiting inter-group differential expression for further investigation. The expression levels of these 11 genes were subsequently measured in cardiomyocytes isolated from the HF group. Our findings revealed that only three genes (CSDC2, PDK4, SMCO4) were detectable in HF cardiomyocytes (Figure 7C). Among these, PDK4 has established functional links to mitochondrial energy metabolism in cardiomyocytes. Given that bulk RNA-seq analyses consistently identified disrupted fatty acid metabolism as a mitochondrial energy metabolism subtype associated with HF, and considering that the roles of CSDC2 and SMCO4 in cardiomyocyte lipid metabolism remain uncharacterized, we further investigated the functional contributions of CSDC2 and SMCO4 to fatty acid metabolism within cardiomyocytes of HF patients.

**Figure 8:**
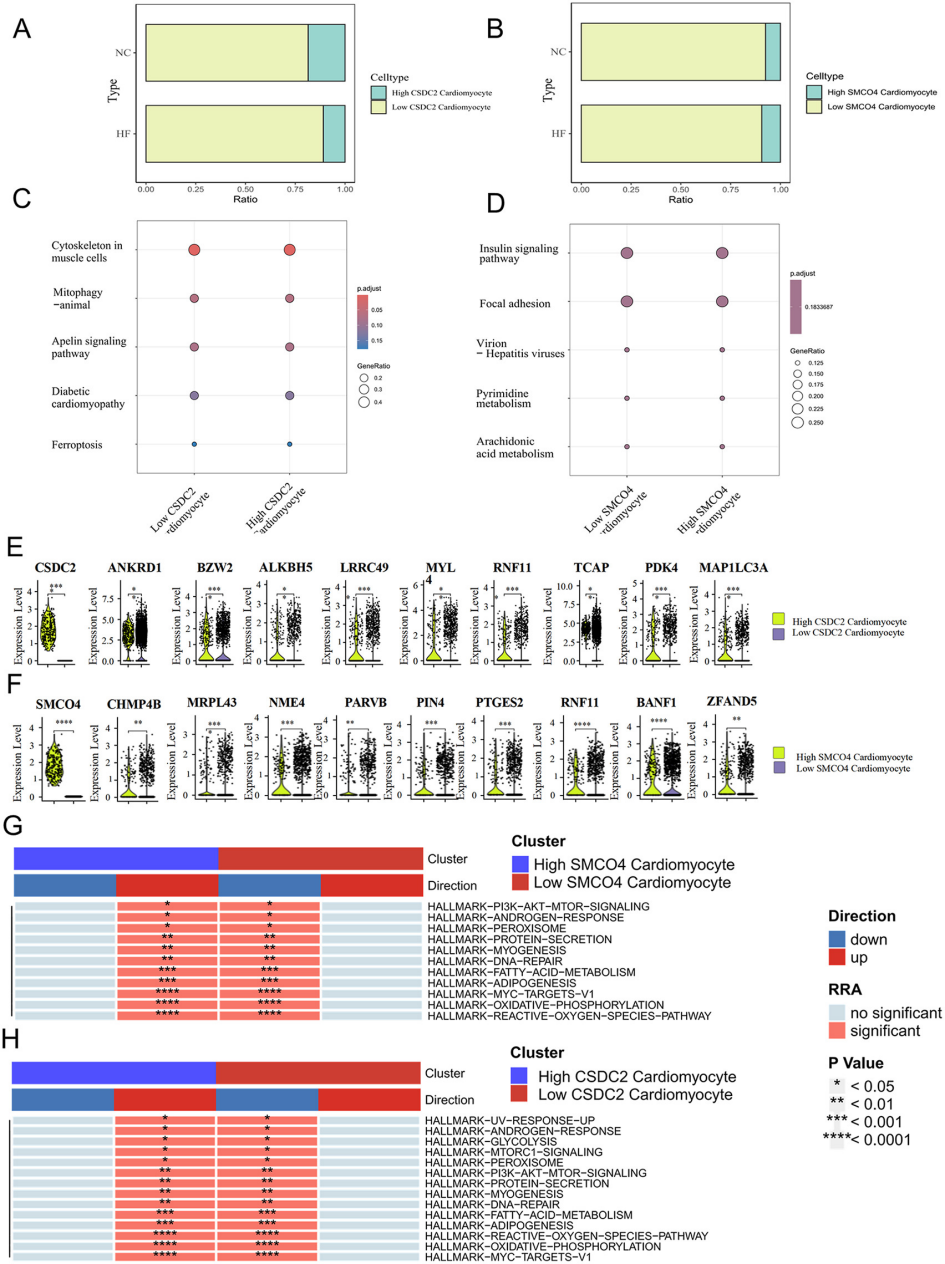
**(A)** Proportional differences between the Cardiomyocytes^CSDC2 high^ subset and the Cardiomyocytes^CSDC2 low^ subset in normal and HF patients' myocardial tissues. **(B)** Proportional differences between the Cardiomyocytes^SMCO4 high^ subset and the Cardiomyocytes^SMCO4 low^ subset in normal and HF patients' myocardial tissues. **(C)** KEGG analysis of differentially expressed genes between the Cardiomyocytes^CSDC2 high^ subset and the Cardiomyocytes^CSDC2 low^ subset. **(D)** KEGG analysis of differentially expressed genes between the Cardiomyocytes^SMCO4 high^ subset and the Cardiomyocytes^SMCO4 low^ subset. **(E)** Lipid metabolism-related genes differentially expressed between the Cardiomyocytes^CSDC2 high^ subset and the Cardiomyocytes^CSDC2 low^ subset. **(F)** Lipid metabolism-related genes differentially expressed between the Cardiomyocytes^SMCO4 high^ subset and the Cardiomyocytes^SMCO4 low^ subset. **(G)** Changes in the enrichment pathways of marker gene sets of differentially expressed genes between the Cardiomyocytes^SMCO4 high^ subset and the Cardiomyocytes^SMCO4 low^ subset. **(H)** Changes in the enrichment pathways of marker gene sets of differentially expressed genes between the Cardiomyocytes^CSDC2 high^ subset and the Cardiomyocytes^CSDC2 low^ subset.

The analysis of cell proportion revealed that there were differences in the expression of CSDC2 and SMCO4 in cardiomyocytes between the normal group and the HF group (Figures 8A,B). The differentially expressed genes between the cardiomyocytes^CSDC2 high^ subset and the cardiomyocytes^CSDC2 low^ subset were mainly concentrated in processes related to myocardial remodeling, such as Cytoskeleton in muscle cells, Mitophagy—animal, Apelin signaling pathway, and Diabetic cardiomyopathy (Figure 8C). Meanwhile, the differentially expressed genes between the cardiomyocytes^SMCO4 high^ subset and the cardiomyocytes^SMCO4 low^ subset were mainly concentrated in processes related to lipid and energy metabolism, such as Insulin signaling pathway, Focal adhesion, Pyrimidine metabolism, and Arachidonic acid metabolism (Figure 8D).

To further clarify the correlation between CSDC2, SMCO4 and fatty acid metabolism, we examined the expression differences of fatty acid metabolism-related genes in the cardiomyocytesCSDC2 high subset, cardiomyocytesCSDC2 low subset, cardiomyocytesSMCO4 high subset, and cardiomyocytesSMCO4 low subset. The results revealed that ANKRD1, BZW2, ALKBH5, LRRC49, MYL, RNF11, TCAP, PDK4, and MAP1LC3A exhibited statistically significant differences (*P* < 0.05) between the cardiomyocytesCSDC2 high subset and cardiomyocytesCSDC2 low subset (Figure 8E). Additionally, CHMP4B, MRPL43, NME4, PARVB, PIN4, PTGES2, RNF11, BANF1, and ZFAND5 displayed statistically significant differences (*P* < 0.05) between the cardiomyocytes^SMCO4 high^ subset and cardiomyocytes^SMCO4 low^ subset (Figure 8F). Further pathway enrichment analysis corroborated these findings: the FATTY-ACID-METABOLISM, ADIPOGENESIS, and OXIDATIVE-PHOSPHORYLATION pathways were significantly upregulated in the cardiomyocytes^SMCO4 high^ subset, while the same pathways (FATTY-ACID-METABOLISM, ADIPOGENESIS, and OXIDATIVE-PHOSPHORYLATION) were also significantly upregulated in the cardiomyocytes^CSDC2 high^ subset (Figures 8G,H). These results suggest that CSDC2 and SMCO4 may influence the progression of HF by modulating lipid metabolism in cardiomyocytes.

## 4 Discussion

HF is a set of systemic syndromes caused by the decompensation of the heart's pumping function, leading to congestion in the pulmonary and systemic circulation. It represents the final stage of many cardiac diseases and continues to pose a challenge to healthcare systems due to its high incidence and mortality rates (24).

In recent years, the treatment of HF has primarily focused on reducing the hemodynamic burden on the heart (25), yet the pathological mechanisms of HF remain unclear. Cardiomyocytes are the main working cell type in the heart and are the most severely affected cells. Therefore, research on the cellular mechanisms and therapeutic strategies targeting cardiomyocytes continues to be the greatest unmet need in cardiology. Cardiomyocytes rely on mitochondrial ATP production to meet high energy demands during contraction/relaxation. Fatty acids serve as the heart's primary fuel, generating 40%–60% of cardiac ATP through oxidation (5). HF initiates pathological metabolic reprogramming characterized by a substrate shift from fatty acid oxidation to glycolysis. This transition reduces ATP yield as glycolysis produces far less energy than oxidative phosphorylation (7), creating an energy deficit that directly contributes to HF progression. Consequently, dysregulation of fatty acid metabolism represents a critical therapeutic target in HF pathophysiology.With the advancement of bioinformatics and multi-omics technologies, leveraging bioinformatics techniques to uncover complex pathogenic mechanisms is of significant importance for the treatment of HF (26).

In this study, we conducted differential gene screening and functional enrichment analysis on the transcriptome sequencing results of myocardial tissue from HF patients in six datasets, including GSE57338, GSE42955, GSE52601, GSE21610, GSE76701, and GSE145154 (which includes one single-cell sequencing transcriptome dataset), using RRA and WGCNA. In this study, RRA's strengths include amplifying biological signals in noisy/heterogeneous data (e.g., integrating microarray results across platforms or incomplete metadata), enabling meta-analysis by consolidating diverse experiments, and retaining meaningful information even with top-ranked elements only, thus enhancing reliability in high-throughput genomic data integration (16). In applications such as HF research, WGCNA's advantages include its ability to identify disease-relevant modules (e.g., gene clusters associated with HF subtypes, clinical traits, or pathological pathways), validate modules via functional enrichment analysis, and link module eigengenes to HF phenotypes (e.g., left ventricular dysfunction or disease progression). This enhances the discovery of potential biomarkers or therapeutic targets by leveraging systems-level gene interactions in complex cardiac diseases (17).

The results indicated that the functional enrichment of RRA differential genes lacked directional characteristics, covering a broad range of cellular life activities. However, the analysis of the key module in the GSE76701 WGCNA dataset showed a certain tendency towards energy metabolism. The GO analysis was primarily enriched in biological functions such as energy production through the oxidation of organic compounds, fatty acid metabolism, mitochondrial genes, long-chain fatty acid transport, and energy reserve metabolism, and in terms of cellular components, it highlighted that the mitochondrial matrix and mitochondrial intermembrane space are the main locations where key genes are primarily localized. The KEGG analysis also enriched in functional pathways related to energy cycle utilization, such as the mTOR signaling pathway, phagosome signaling pathway, and lysosome signaling pathway. After taking the intersection of the key gene modules from the RRA analysis and the WGCNA analysis, 14 key genes were identified.

Research has found that when HF occurs, there is a dramatic change in myocardial energy metabolism, with a loss of metabolic flexibility (27, 28) and insufficient ATP production (29, 30). A normal heart requires a large amount of ATP, 95% of which is derived from oxidative phosphorylation in mitochondria, with the remaining 5% coming from glycolysis. After the onset of HF, cardiac metabolism is reprogrammed to favor glycolysis as the energy substrate, shifting from fatty acid metabolism to glucose metabolism. The energy output efficiency of glycolysis is much lower than that of oxidative phosphorylation, resulting in an energy deficiency. Currently, there is no consensus on how substrate metabolism is transformed after HF and the exact issues with each metabolic pathway (7). However, it is generally believed that the energy deficiency is attributed to impaired mitochondrial oxidative phosphorylation and changes in ATP substrate sources.

Fatty acids constitute the primary fuel for the heart, with fatty acid oxidation accounting for 40%–60% of cardiac ATP production. Fatty acids are supplied to the myocardium through three principal pathways: (1) albumin-bound free fatty acids (FFAs) from systemic circulation, (2) triglyceride (TAG) delivery via chylomicrons and very-low-density lipoproteins (VLDL) hydrolyzed by lipoprotein lipase (31–33), and (3) mobilization from intracellular TAG stores within cardiomyocytes (34). FFAs enter cardiomyocytes through facilitated diffusion or via fatty acid transport proteins and fatty acid translocase (35).

Within the cytosol, approximately 90% of fatty acids undergo mitochondrial shuttling for oxidation, while the remaining 10% are esterified into TAG for cellular storage as reserve substrates (36). Mitochondrial fatty acid oxidation requires sequential activation and transport steps (37): Cytosolic FFAs are first esterified to fatty acyl-CoA, which is then converted to long-chain acylcarnitine by carnitine palmitoyltransferase-1 (CPT-1) at the outer mitochondrial membrane (37). After traversing the inner membrane via carnitine-acylcarnitine translocase, CPT2 reconverts acylcarnitine to fatty acyl-CoA in the matrix. Subsequent β-oxidation generates acetyl-CoA, which enters the TCA cycle to produce NADH and FADH_2_ (38). These electron donors fuel the electron transport chain to drive oxidative phosphorylation—the primary ATP-generating pathway in cells. Consequently, targeting fatty acid metabolism to sustain oxidative phosphorylation capacity represents a critical therapeutic strategy in HF.

In this study, after validation via qPCR, it was confirmed that 12 key differentially expressed genes indeed showed differential expression in Ang II-induced hypertrophic cardiomyocytes. To experimentally validate the association between the 14 key genes and cardiomyocytes, we selected 11 intergroup differentially expressed genes for further investigation. Leveraging the GSE145154 single-cell sequencing dataset derived from ventricular tissue, our findings revealed that only three genes (CSDC2, PDK4, SMCO4) were detectable in HF cardiomyocytes. Given that extensive RNA-seq analyses have consistently identified dysregulated fatty acid metabolism as a mitochondrial energy metabolic subtype associated with HF, and considering the undetermined roles of CSDC2 and SMCO4 in lipid metabolism within cardiomyocytes, we further explored their functional contributions to fatty acid metabolism in cardiomyocytes of HF patients.

CSDC2, a cardiac-enriched RNA binding protein downregulated in failing hearts, is identified as a critical regulator of cardio-metabolic stress. Consistent with our findings, Jared M McLendon et al. also identified through bioinformatic analyses that CSDC2 directly participates in regulating fatty acid metabolism pathways, as well as ion channels and sarcomere gene networks. Validation using Csdc2-knockout (KO) mice revealed that, after 10 weeks of high-fat diet feeding, Csdc2-KO mice exhibited a 20% greater weight gain than wildtype littermates (*p* = 0.04, *n* = 13–18) and developed insulin resistance (*p* < 0.01). Furthermore, Csdc2-KO mice displayed larger infarcts, ventricular remodeling, and systolic dysfunction following microsurgery-induced MI (39).

SMCO4 is a single-pass transmembrane protein with a coiled-coil domain. Studies by Nicholas A Wachowski et al. demonstrated that knockdown of SMCO4 expression in pancreatic β-cells significantly enhances insulin secretion (40). Insulin plays a crucial role in cardiac fatty acid metabolism and energy metabolism, influencing the utilization of fatty acids and glucose by regulating the metabolic flexibility of cardiomyocytes (41). Under conditions of elevated insulin levels, the heart tends to increase glucose oxidation while inhibiting fatty acid oxidation, thereby affecting cardiac mechanical efficiency and function (42). These findings are consistent with our study, where we observed that the Cardiomyocytes^SMCO4 low^ subset exhibit inhibition of fatty acid metabolic pathways. This suggests that despite the current paucity of similar studies, SMCO4 still demonstrates potential as a therapeutic target for HF. Future investigations into its underlying mechanisms are warranted to facilitate the prevention and treatment of HF.

This study has certain limitations. First, it remains imperative to objectively evaluate its alignment with and distinctions from prior large-scale transcriptomic meta-analyses. For example, Ramirez Flores et al. consolidated 16 studies (*n* = 916) to validate consensus transcriptional signatures in end-stage HF and developed the open platform ReHeat for biomarker discovery and therapeutic target identification (43). By contrast, although we included fewer datasets, these datasets were not entirely overlapping. We utilized multiple algorithms to analyze and identify key hub genes associated with HF, introduced RT-qPCR and single-cell sequencing datasets to validate the results, and ultimately identified SMCO4 and CSDC2 as key genes regulating fatty acid metabolism and influencing HF progression. Nonetheless, constrained by the limited sample size (*n* = 422), the statistical sensitivity of this study may be suboptimal, necessitating future expansion of cohorts to validate the generalizability of the metabolic modules. Further validation of the roles of CSDC2 and SMCO4 in fatty acid metabolism still requires basic experimental verification.

Additionally, this study used a primary neonatal rat cardiomyocyte hypertrophy model to validate the results. Although such cell models are classic and commonly used in HF research (20, 44), the transcriptomic characteristics of primary neonatal rat cardiomyocytes are also difficult to be fully consistent with those of adult human cardiomyocytes. Although single-cell sequencing datasets were introduced to address this issue, it still indicates that further validation is required in future studies.

Another limitation of our study is the absence of formal sensitivity analyses, such as leave-one-out or bootstrap-based testing, to further assess the robustness of RRA-derived results. Although the inclusion of multiple independent datasets enhances the credibility of consistently identified DEGs, future work will incorporate systematic sensitivity analyses to quantitatively evaluate the impact of individual datasets and strengthen the reliability of integrative meta-analysis approaches.

## 5 Conclusion

Integrated transcriptomic analysis identified 12 key genes linked to HF, which were validated in a hypertrophy model. Single-cell data showed SMCO4 and CSDC2 are specifically expressed in cardiomyocytes and regulate fatty acid metabolism. This suggests SMCO4 and CSDC2 contribute to HF by altering fatty acid metabolism in heart cells, revealing new disease mechanisms.

## Data Availability

The original contributions presented in the study are included in the article/Supplementary Material, further inquiries can be directed to the corresponding authors.
