# Supplementary figures and images for: Analysis and validation of characteristic genes in RNA sequencing datasets from heart failure patients based on multiple algorithms
Source: Front Cardiovasc Med. 2025 Aug 26;12:1559429. doi: 10.3389/fcvm.2025.1559429 (PMC12417514; doi:10.3389/fcvm.2025.1559429)

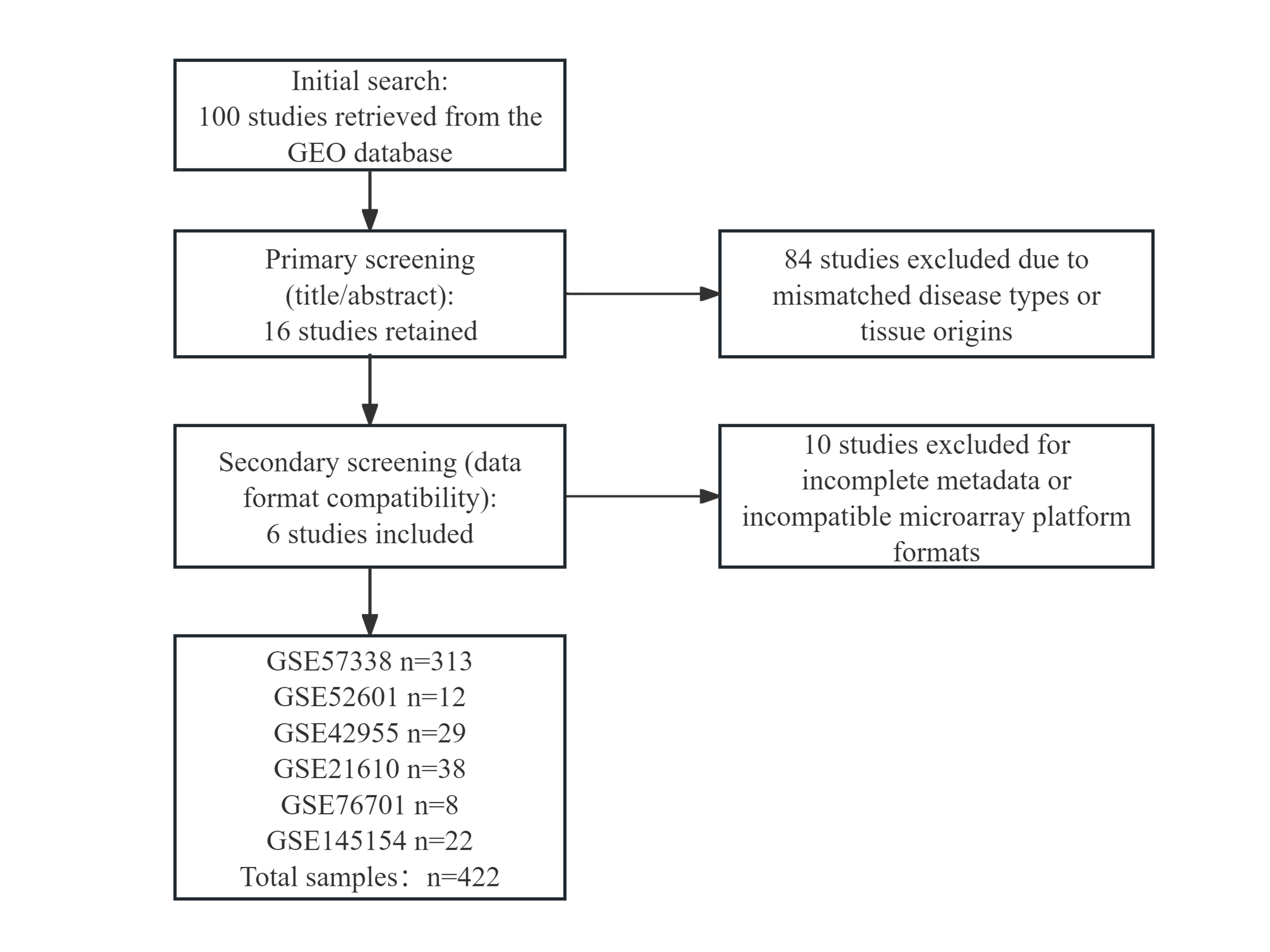

Supplement: Supplementary file 1 [file Image1.tif]
