# Supplementary material for: Analysis and validation of characteristic genes in RNA sequencing datasets from heart failure patients based on multiple algorithms
Source: Front Cardiovasc Med. 2025 Aug 26;12:1559429. doi: 10.3389/fcvm.2025.1559429 (PMC12417514; doi:10.3389/fcvm.2025.1559429)
Supplement: Supplementary file 2 [file Table1.docx]

Table S1 Clinical Characteristics of GEO Datasets

| GEO ID | Sample Type | Age (years, Mean ± SD) | Sex (Male/Female, n/%) | Disease Type (n/%) |
| --- | --- | --- | --- | --- |
| GSE57338 | Heart failure | 55.5±11.6 | 144(81.3%)/33(18.6%) | DCM (53.6%)/ ICM (46.4%) |
|  | Normal | 49.36±15.0 | 73(41.2%)/63(35.5%) | / |
| GSE52601 | Heart failure | 61.5±8.6 | 6(75%)/2(25%) | DCM (50%)/ ICM (50%) |
|  | Normal | 39.5±26.4 | 4(100%)/0(0%) | / |
| GSE42955 | Heart failure | / | 24(100%)/0(0%) | DCM (50%)/ ICM (50%) |
|  | Normal | / | 5(100%)/0(0%) | / |
| GSE21610 | Heart failure | 51.3±12.8 | 27(93.1%)/2(6.9%) | DCM (70%)/ ICM (30%) |
|  | Normal | 29.0±17.4 | 6(75%)/2(25%) | / |
| GSE76701 | Heart failure | 57.0±3.6 | 3(75%)/1(25%) | ICM (100%) |
|  | Normal | 57.5±12.5 | 0(0%)/4(100%) | / |
| GSE145154 | Heart failure | / | / | DCM (44.4%)/ ICM (55.6%) |
|  | Normal | / | / | / |

Tips:DCM, Idiopathic Dilated Cardiomyopathy; ICM. ischemic cardiomyopathy;
